# Planting molecular functions in an ecological context with *Arabidopsis thaliana*

**DOI:** 10.7554/eLife.06100

**Published:** 2015-03-25

**Authors:** Ute Krämer

**Affiliations:** Department of Plant Physiology, Ruhr University Bochum, Bochum, Germany

**Keywords:** the natural history of model organisms, natural history, Arabidopsis relatives, Arabidopsis

## Abstract

The vascular plant *Arabidopsis thaliana* is a central genetic model and universal reference organism in plant and crop science. The successful integration of different fields of research in the study of *A. thaliana* has made a large contribution to our molecular understanding of key concepts in biology. The availability and active development of experimental tools and resources, in combination with the accessibility of a wealth of cumulatively acquired knowledge about this plant, support the most advanced systems biology approaches among all land plants. Research in molecular ecology and evolution has also brought the natural history of *A. thaliana* into the limelight. This article showcases our current knowledge of the natural history of *A. thaliana* from the perspective of the most closely related plant species, providing an evolutionary framework for interpreting novel findings and for developing new hypotheses based on our knowledge of this plant.

**DOI:** http://dx.doi.org/10.7554/eLife.06100.001

## Introduction

**Figure 1.**
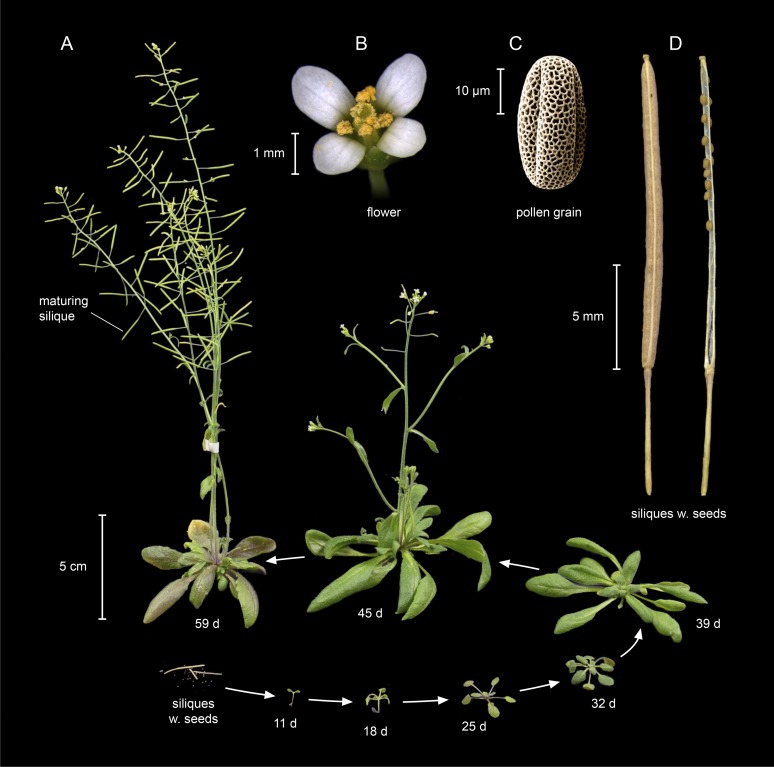
Life cycle of *Arabidopsis thaliana*. (**A**) *A. thaliana* of the accession Columbia (Col) at different stages of its life cycle, from seed (bottom left) to seedling (11 days), to vegetative growth (39 days), and to reproductive growth (45 days). Photographs of (**B**) a flower, (**C**) a pollen grain (scanning electron micrograph), and (**D**) mature siliques (seed pods; left: closed; right: open with a few remaining unshattered seeds) at higher magnification. Image credits: **B** and **C**, Maria Bernal and Peter Huijser; other photographs, Ines Kubigsteltig and Klaus Hagemann. **DOI:** http://dx.doi.org/10.7554/eLife.06100.002

*Arabidopsis thaliana* is a small, annual or winter annual, rosette plant (Figure 1). It belongs to the taxonomic family of the Brassicaceae in the eudicotyledonous group of angiosperm vascular plants (see Box 1 for a glossary of specialist terms used in this article). This family also includes morphologically diverse but closely related oilseed crops, vegetables and spice plants, for example rapeseed, brussel sprouts, various cabbages, cauliflower, garden radish and mustard. First described by the physician Johannes Thal in the Harz Mountains of Northern Germany in 1577, *A. thaliana* featured in the *Species Plantarum II* published by Linnaeus in 1753, and it received its present name, *Arabidopsis thaliana* (L.) Heynh, from Gustav Heynhold in 1842 (Kück, 2005). Friedrich Laibach was the first researcher to publish an influential cytogenetic study on *A. thaliana* in 1907, to foster genetics in the 1940s, and to systematically collect accessions in the wild after emphasizing the extent of intraspecific phenotypic variation in *A. thaliana* in the 1950s (Somerville and Koornneef, 2002; Koornneef and Meinke, 2010).

10.7554/eLife.06100.003

Box 1. **Glossary**

**Allopolyploidy:** Following the hybridization of two different species, this term describes when two (or more) chromosome sets from each parent are maintained in the genome.

**Autopolyploidy:** Polyploidy arising from the multiplication of chromosome sets in a genome, often due to the formation of unreduced gametes in an aberrant meiosis.

**Calcareous outcrops:** Rocky areas with predominantly calcium carbonate-rich minerals, sparse vegetation, soils of high pH with poor biovailability of some nutrients and poor water-holding capacity.

**Commensal:** An organism that lives in close association with a host organism, from which it benefits—usually by receiving nutrients—without harming the host or being beneficial to it.

**Effector-triggered immunity:** A form of plant innate immunity mediated by plant NLR proteins (see below) of diverse recognition specificities, encoded by *Resistance* (*R*) genes organized in recombinogenic, but somatically stable, gene clusters. Each R protein detects a pathogen-derived effector molecule or its action on a host target protein.

**Eudicotyledon:** Angiosperm (flowering) plant species characterized by two cotyledons (embryonic leaves) and other morphological characteristics.

**Glucosinolates:** A group of secondary metabolites characteristic of the plant order Brassicales, including the Brassicaceae family.

**Induced systemic resistance:** Systemic resistance to infection by a broad spectrum of pathogens and to attack by herbivores. Its induction is triggered locally (usually in the root) by the presence of plant-associated beneficial microbes.

**Metal hyperaccumulation:** The accumulation in above-ground plant structures (usually leaves) of metals or metalloids at concentrations far higher than in non-accumulator plants at the same site. A rare trait found in ∼0.2% of angiosperms that acts as an elemental defense against biotic stress.

**Molecular pattern-triggered immunity:** A form of plant innate immunity mediated by transmembrane pattern recognition receptors that detect evolutionarily conserved molecules, called cell damage-associated or microbe-associated molecular patterns, usually on the plant cell surface.

**NLR proteins:** NLR (nucleotide-binding domain leucine-rich repeat) proteins function in plant effector triggered immunity. Also denotes a family of proteins found subsequently to act in animal innate immunity. All NLR proteins share a similar three-domain architecture.

**Phenology:** The timing, or the study, of periodic life cycle events in organisms in conjunction with seasonal cycles and/or inter-annual climatic variation.

**Photosynthate:** A product of photosynthesis, that is, an energy-rich carbon-containing molecule (most commonly a sugar).

**Plant growth-promoting rhizobacteria:** Beneficial bacteria that enhance plant growth and biomass production and live in association with plant roots in natural environments.

**Synanthropy:** Occurrence of an organism in association with human settlement, where human activities directly or indirectly generate favorable environmental conditions for the organism.

**Ultramafic soils:** Infertile, nutrient poor soils derived from so-called serpentine minerals, which are common in zones of tectonic activity.

**DOI:** http://dx.doi.org/10.7554/eLife.06100.003

The popularity of *A. thaliana* as a model organism took off in the 1980s, and then rapidly gained momentum when researchers successfully began to combine genetics with powerful molecular biology methods. The Arabidopsis reference genome sequence was published as the first nuclear genome of a flowering plant in 2000 (http://www.arabidopsis.org/) (Arabidopsis Genome Initiative, 2000; Somerville and Koornneef, 2002; Koornneef and Meinke, 2010). The ease and speed with which experiments can be conducted on *A. thaliana* has allowed enormous fundamental progress in our knowledge of the molecular principles of plant development, cell biology, metabolism, physiology, genetics and epigenetics (http://www.arabidopsisbook.org/). The many uses of *Arabidopsis* as the universal reference plant continue to expand, particularly in the field of systems biology (Brady et al., 2007; Endo et al., 2014), and more recently provide a means of placing molecular functions into an ecological and evolutionary context in natural environments (Tian et al., 2003; Mitchell-Olds and Schmitt, 2006; Alonso-Blanco et al., 2009; Atwell et al., 2010; Bergelson and Roux, 2010; Fournier-Level et al., 2011; Hancock et al., 2011; Züst et al., 2012).

**Figure 2.**
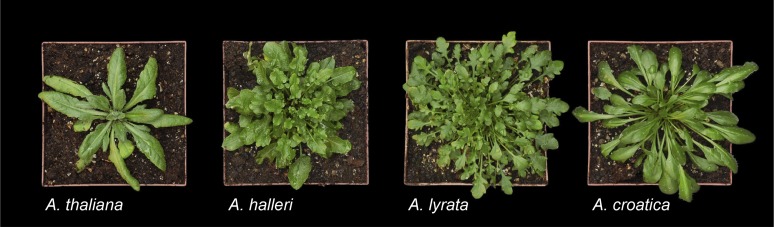
*A. thaliana* and a subset of species from its sister clade. From left to right: *A. thaliana* (Col), *A. halleri* (ssp. *halleri*; individual Lan5, Langelsheim, Harz, Germany), *A. lyrata* (ssp. *lyrata*; selfing accession Great Lakes, North America), and *A. croatica* (Baške Oštarje/Ljubičko Brdo, Croatia). *A. thaliana* was grown from seed to early reproductive stage, and the other species were propagated vegetatively and grown for 3–6 months. The individuals shown here do not reflect the large within-species morphological diversity, particularly in leaf shape, among different accessions of *A. halleri* and *A. lyrata*. Image credit: Ute Krämer and Klaus Hagemann. **DOI:** http://dx.doi.org/10.7554/eLife.06100.004

Many of the traits that render *A. thaliana* an attractive model plant also constitute adaptations that reflect the ecological niche occupied by this species, such as the short generation time and the ability to self-fertilize (selfing). Indeed, the natural history of *A. thaliana* differs notably from that of the species in its sister group of the most closely related existing plant species, which includes *Arabidopsis lyrata*, *Arabidopsis arenosa* and *Arabidopsis croatica*, and the three species *Arabidopsis halleri*, *Arabidopsis cebennensis* and *Arabidopsis pedemontana* (Koch and Matschinger, 2007; Hohmann et al., 2014) (Figure 2). Within these six evolutionary lineages, a number of phylogenetically (as yet) unresolved taxa have been described, as have auto- and allopolyploidization events (see Glossary). The divergence time of the common ancestor of these species and the *A. thaliana* lineage was estimated at around 5 million years ago (Mya) (Koch et al., 2000), with upper and lower bounds of 17.9 and 3.1 Mya (Koch et al., 2000; Beilstein et al., 2010; Ossowski et al., 2010). Thus, divergence occurred immediately before or during the transition from a warm period to progressively colder climates, followed by rapid glacial cycles from 3 Mya until the latest glacial maximum about 18,000 years ago. There has even been a natural allopolyploidization event between *A. arenosa* and *A. thaliana* 10,000–300,000 years ago, giving rise to *Arabidopsis suecica* (Sall et al., 2003; Jakobsson et al., 2006).

Work in recent years has increasingly emphasized the relevance of conducting experiments on *A. thaliana* in natural or semi-natural settings (Wilczek et al., 2009; Brachi et al., 2010; Chiang et al., 2013; Karasov et al., 2014). This important development will benefit from a more detailed knowledge of the unique and shared features of its natural and evolutionary history, as well as of the natural environments that host populations of *A. thaliana*.

## The natural history of *A. thaliana* in an evolutionary and ecological context

**Figure 3.**
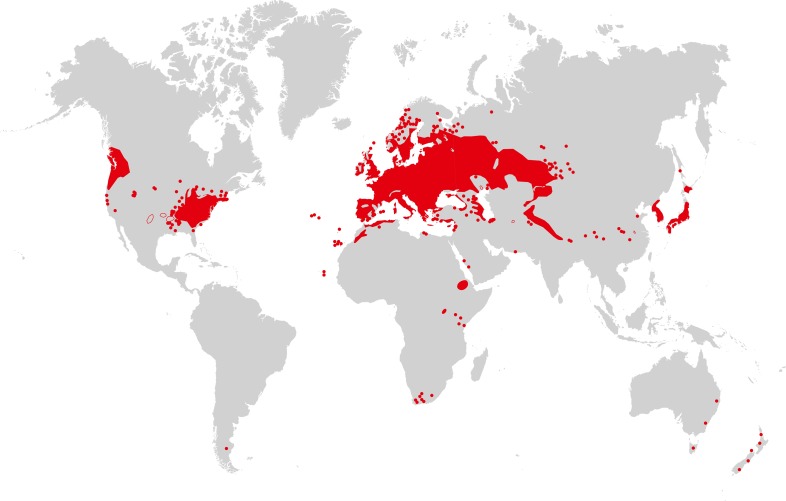
Map of *A. thaliana* worldwide distribution. Areas colored in red correspond to the continuous distribution of *A. thaliana*; red circles mark additional sites. This map is based on a partial map kindly provided by Matthias Hoffmann (personal communication, November 2014), with manual additions to the southern hemisphere (Bresinsky et al., 2008). Image credit: Ute Krämer and Klaus Hagemann. **DOI:** http://dx.doi.org/10.7554/eLife.06100.005

*Arabidopsis thaliana* is recognized as native to Western Eurasia (Figure 3). The species is a colonizer and pioneer plant of disturbed, poor, stony or shallow soils, and it can also be found in nutrient-poor, often sandy, meadow and forest habitats (Mitchell-Olds and Schmitt, 2006). Similarly, the species closely related to *A. thaliana* are able to colonize bare rocky habitats. Sequence polymorphism data provide evidence for a historical expansion of the *A. thaliana* species population (Nordborg et al., 2005; Schmid et al., 2005), thus differing from the known demographic histories of the non-hybrid species in its sister group in the *Arabidopsis* genus (Wright et al., 2003; Heidel et al., 2010). It has been suggested that *A. thaliana* re-colonized Central Europe from glacial refugia in the Caucasus or Balkans between ∼10,000 and 5000 years ago, possibly accompanying the spread of human farming from the Near East (Francois et al., 2008). There is also clear evidence for additional glacial refugia for *A. thaliana* in the Mediterranean region, including the North African Atlas Mountains (Brennan et al., 2014), and for additional migration (for example, from the Iberian peninsula in a northeastern direction [Sharbel et al., 2000; Pico et al., 2008]), as well as for population structure reflecting isolation by distance in Eurasia (Nordborg et al., 2005). This reflects an evolutionary history of vast climatic and environmental fluctuations, likely including a series of alternating phases of population size contraction and expansion with migration and admixture following glacial cycles (Beck et al., 2008). In the past few 100 years, *A. thaliana* has further expanded its geographic and climatic range in synanthropy (see Glossary), migrating to and across North America, as well as southward in Africa, and more recently to East Asia (see Figure 3). The present climatic and geographic range of *A. thaliana* is larger than that of any of its close relatives, which have either not expanded into warmer climates or are local endemics (Koch and Matschinger, 2007; Hohmann et al., 2014).

In addition to—and likely permitting—the rapid expansion of its range, *A. thaliana* has undergone transitions to reproduction by self-fertilization (selfing). Estimates place these transitions as having occurred, based on different approaches, at around 0.4 Mya or around ≥1 Mya (Bechsgaard et al., 2006; Tang et al., 2007). By contrast, most species closely related to *A. thaliana* are either predominantly or strictly outcrossing (the latter if species are strongly self-incompatible). In addition to selfing, *A. thaliana* is an annual plant with a rapid life cycle that can be as short as 6–8 weeks in some accessions, whereas most of its close relatives are biannuals or herbaceous perennials (Al-Shehbaz and O'Kane, 2002) (see Figures 1, 2). Vegetative *A. thaliana* plants are smaller in size than most of its close relatives, and their survival and reproduction are comparably robust in a variety of environments. The leaves of *A. thaliana* are positioned to form a more compact rosette, and inflorescences are more erect and elongated, than in its close relative *A. halleri*. These morphological differences are likely to constitute adaptations to differing environments. By comparison to its sister group of species, the flowering of *A. thaliana* is highly synchronized, with the formation of a larger number of siliques (seed pods) per plant, each containing a higher, or far higher, number of seeds that are smaller in size compared to those of its relatives. Seeds of *A. thaliana* are long-lived and highly viable by comparison to those of *A. halleri* and *A. lyrata* (Ute Krämer, personal observation). These features of *A. thaliana* render it ideal for mutagenesis, genetics, and for the identification, selection and propagation of mutant or transgenic lines, as well as for stock maintenance.

**Figure 4.**
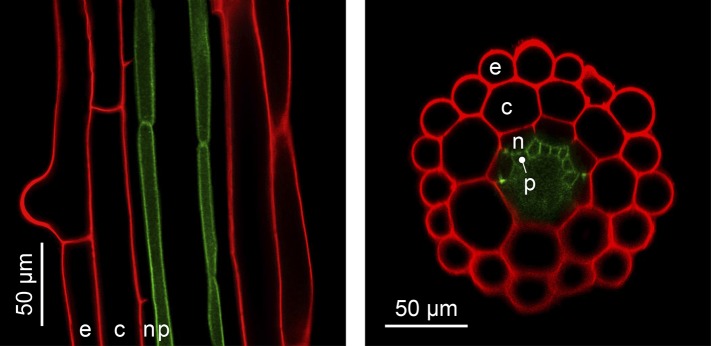
The simple anatomy of *A. thaliana* roots. Longitudinal (left) and transverse (right) confocal sections of *A. thaliana* roots. Green fluorescence highlights the plasma membrane of the pericycle cells of an *A. thaliana hma2hma4* double mutant line (Sinclair et al., 2007) (accession Wassilewskija). Red fluorescence of propidium iodide (PI) as a stain is overlaid to visualize cell walls. The root cell layers (consecutive outward to inward) are: epidermis (e), cortex (c), endodermis (n) and pericycle (p). Note that the Casparian Strip surrounding the endodermis cells forms an apoplastic diffusion barrier (Roppolo et al., 2011) that blocks the movement of PI further inward. Image credit: Ute Krämer and Scott A. Sinclair. **DOI:** http://dx.doi.org/10.7554/eLife.06100.006

The complexity of organ architecture appears to be reduced in *A. thaliana*. Notably, its roots mostly contain only a single layer of each specialized cell type, a feature that is optimal for *in vivo* imaging (Figure 4) (De Lucas and Brady, 2013). This organization differs from the roots of its close relative *A. halleri*, which contain two additional cell layers (Hanikenne et al., 2008), and is in stark contrast to root tissues composed of multiple cell layers, as found in many other plants. The metabolic cost of building an *A. thaliana* root is evidently minimal, and this is likely to have ecological implications which have yet to be determined.

The ancestral number of chromosomes in the Brassicaceae (*n* = 8) is conserved in *A. thaliana*'s closest relatives. By comparison, the genome of the diploid *A. thaliana* is rearranged, with two sets of *n* = 5 chromosomes. This is associated with a pronounced reduction in genome size in *A. thaliana*, down to 157 Mbp (Bennett et al., 2003) (reference genome assembly of 135 Mbp)—one of the smallest known genomes among flowering plants. For comparison, nuclear genome sizes of its closest relatives are larger, mostly due to a higher abundance of transposable elements in euchromatic regions, with 207 Mbp for the reference assembly of the nuclear genome of *A. lyrata* (Hu et al., 2011), and about 260 Mbp estimated for *A. halleri* (Johnston et al., 2005; Peer et al., 2006; Hohmann et al., 2014). These properties of *A. thaliana* have greatly facilitated the sequencing, assembly and annotation of its genome, as well as molecular genetics and genomics approaches.

All of these characteristic features of *A. thaliana* are likely to constitute adaptations that jointly define the ecological niche of the species and reflect an ecological strategy that has apparently been highly successful in recent evolutionary history.

## Plant development and its environmental control

Classical molecular genetics approaches have been used in *A. thaliana* to dissect the patterning and development of flowers (Meyerowitz et al., 1991), embryos (Mayer et al., 1991), leaves and roots (Di Laurenzio et al., 1996), and of the maintenance of stem cell niches at the plant's growing tips. These stem cells fuel the ongoing (indeterminate) growth that is characteristic of plants (Clark et al., 1997; Lenhard et al., 2001). The biology of *A. thaliana* is rich in temperature- and light-dependent regulation, some of which might be triggered by photosynthates (see Glossary) rather than by light itself (Haydon et al., 2013). Important work has investigated the molecular networks that mediate environmentally controlled developmental switches in *A. thaliana*, as well as natural intraspecific variation in phenology (see Glossary). Examples include the light-regulated development of seedlings (photomorphogenesis) (Deng et al., 1992; Nemhauser, 2008), the transition from vegetative to reproductive development, also termed flowering time control (Simpson and Dean, 2002; Valverde et al., 2004; Andres and Coupland, 2012), and seed dormancy and germination control (Bentsink et al., 2006; Graeber et al., 2012), as well as natural variation in these traits (Brachi et al., 2010; Chiang et al., 2013). The need for developmental transitions to be timed accurately and robustly is reflected in the emerging complex, multi-layered epigenetic regulation of some key loci, for example the flowering control gene *Flowering Locus C* (*FLC*) (Simpson and Dean, 2002; Csorba et al., 2014). Such complex regulation in *A. thaliana* may have evolved in a step-wise fashion alongside recurrent climatic fluctuations encountered by *A. thaliana* during its evolutionary history. Nevertheless, contemporary global warming appears to proceed faster than the local adaptation of *A. thaliana* populations (Wilczek et al., 2014), based on the reproductive fitness of plants grown at locations across the European distribution range from stored seeds, which were collected in different geographic regions between 4 and 70 years earlier.

Phenotypic plasticity and utilizing limited local resources in bare habitats for rapid and ample reproduction appear to be characteristics of the life history of *A. thaliana*. The plant also seemingly evades periodic or episodic environmental adversities, including between-plant competition, *via* a rapid life cycle. Consequently, the adjustment of developmental timing to environmental conditions is likely to be of major importance in the phenotypic plasticity of *A*. *thaliana* (Martinez et al., 2004; Riboni et al., 2014), and an apparent target of selection (Horton et al., 2012). This might also contribute further to the observed regulatory complexity of flowering time control (see preceding paragraph).

## Other interactions with the environment

In contrast to the vast majority of flowering plants, the roots of the Brassicaceae generally do not undergo mycorrhizal or rhizobial symbiosis. This is likely to make *A. thaliana* comparably independent of specific soil microbes or microbiomes, which may be advantageous for a colonizer of recently disturbed and exposed soils. Additionally, it allows the plant to commit all energy expenditure to its own growth and reproduction, but heightens the demands on the endogenous machinery for nutrient acquisition and efficiency, and for protecting roots from environmental stress. Some of the central molecular components of these pathways have been identified in *A. thaliana* before their discovery in other kingdoms of life, for example, the biosynthesis of low-molecular-weight chelators for micronutrient metal cations through nicotianamine synthase (Trampczynska et al., 2006; Schuler et al., 2012), and for both micronutrient and non-essential toxic metal cations through phytochelatin synthase (Clemens, 2006; Tennstedt et al., 2009). Moreover, the effective and balanced acquisition of adequate quantities of inorganic nutrients, such as phosphate, nitrogen and iron, and their efficient utilization by *A. thaliana* plants have been observed and characterized at the molecular level (Abel, 2011; Hindt and Guerinot, 2012; Araya et al., 2014). When natural accessions of *A. thaliana* are cultivated under uniform controlled conditions, genetically controlled within-species variation is observed in leaf nutrient concentrations (Atwell et al., 2010), which appears to be adaptive at least in some cases (Poormohammad Kiani et al., 2012).

Key traits of *A. thaliana* are the timing of germination to coincide with favorable environmental conditions, a short life cycle, and the ability to tolerate unfavorably hot or dry conditions in the form of de-hydrated seeds. Consequently, we would expect stress tolerance to be less elaborate in *A. thaliana* than in some other plants. Indeed, despite a very wide geographic distribution range, there are no reports of *A. thaliana* accessions that exhibit extreme physiological traits, different from its sister group of plant species. As one out of a total of ∼500 known metal hyperaccumulator plant species (see Glossary), *A. halleri* accumulates extraordinarily high concentrations of zinc and cadmium in leaves where these metals act as a so-called elemental defense against biotic stress (Boyd, 2010; Krämer, 2010). In addition, several species in the sister clade of *A. thaliana* are well-known members of plant communities found in extreme habitats. Calamine soils rich in zinc, cadmium and lead—commonly as a result of anthropogenic pollution—are readily colonized by *A. halleri* (Ernst, 1974; Krämer, 2010), and occasionally by *A. arenosa* (Przedpełska and Wierzbicka, 2007). In addition, there are some natural populations of *A. arenosa* and *A. lyrata* on nutrient-poor ultramafic soils (Holubover serpentinite, 2002; Turner et al., 2010), which contain high levels of nickel and magnesium originating from the local bedrock (see Glossary). Finally, populations of *A. lyrata* (Al-Shehbaz and O'Kane, 2002) and *A. croatica* (Plants, 2014) characteristically inhabit calcareous outcrops (see Glossary).

Above-ground (Vorholt, 2012; Horton et al., 2014) and below-ground organs of *A. thaliana*, and their immediate vicinity, provide habitats for microbial communities, which appear to be more strongly influenced by soil type than by plant genotype (Bulgarelli et al., 2012; Lundberg et al., 2012; Schlaeppi et al., 2014). These microbial communities can include commensals (see Glossary), as well as various naturally occurring viral, bacterial, fungal and oomycete pathogens. Arabidopsis possesses a wealth of general molecular pattern-triggered innate immunity responses (Schwessinger and Ronald, 2012) and more-specific, effector-triggered and *Resistance* (*R*) gene-dependent, defenses against microbes (Cui et al., 2014) (see Glossary). Their elucidation has extensively contributed to our understanding of plant defenses against pathogens. By comparison, we understand far less about how pathogens shape genetic diversity in *A. thaliana* natural populations (Karasov et al., 2014) and about the establishment and maintenance of beneficial interactions of *A. thaliana* with microbes, such as plant growth-promoting rhizobacteria and microbes that trigger induced systemic resistance (Lugtenberg and Kamilova, 2009; Pieterse et al., 2014) (see Glossary). Once *A. thaliana* has initiated reproductive development, reproduction is favored over defense (Winter et al., 2011). This is consistent with the general ecological strategy of *A. thaliana* towards an escape from stress through resource allocation that prioritizes reproduction, followed by a rapid conclusion of the life cycle, which differs from its longer-lived relatives. Similarities have been noted between plant defenses and animal innate immunity. These relate primarily to the domain architectures of NLR (nucleotide-binding domain leucine-rich repeat) protein receptors (see Glossary) that function in the detection of biotic attack, and the programmed cell death that occurs as a host defense response (Maekawa et al., 2011).

*Arabidopsis* possesses effective defenses against herbivory, for example by insects and snails, with an important role for secondary metabolites. Most prominently, a class of sulfur-rich compounds, called glucosinolates (see Glossary), are converted to pungent and toxic breakdown products in damaged tissues (Halkier and Gershenzon, 2006). These compounds are agronomically important determinants of taste and are receiving much attention as health-protective, anti-cancer components of ‘functional foods’ (Sonderby et al., 2010). Upon leaf wounding, the systemic activation of defenses in distant leaves involves the long-distance movement of electrical surface potential changes at velocities between 43 and 150 µm s^−1^ involving ionotropic glutamate receptor (iGluR)-related membrane proteins (Mousavi et al., 2013). Strikingly, this bears some similarity to the role of vertebrate iGluRs in fast excitatory synaptic transmission in the central nervous system. The fast systemic movement of electrical signals turns out to be of broader importance in plant responses to the environment. For example, upon exposure of *A. thaliana* to salt stress, a systemic signal moves from root to shoot in the form of a Ca^2+^ wave at a velocity of 400 µm s^−1^, requiring the vacuolar ion channel TPC1 (Two Pore Ca^2+^ Channel protein 1) (Choi et al., 2014). These findings underpin the central importance of *A. thaliana* as a model for obtaining molecular insights into fundamentally novel aspects of plant biology.

## Metabolism in the context of natural history

Metabolic properties are noteworthy components of natural history because they are likely to facilitate, constrain or even prevent specific adaptations in a given lineage sharing them. With respect to nutrient ions, plants of the Brassicaceae family were classified as calciotrophic, based on their ability to store comparably large quantities of dissolved calcium (Ca^2+^) (Kinzel, 1982). This is physiologically challenging because cytosolic free Ca^2+^ levels have to be maintained at very low (<0.1 µM) resting levels continuously in order to allow for Ca^2+^-mediated signaling to occur (Choi et al., 2014). By contrast, calciophobic plants contain Ca in the form of insoluble precipitates, mostly oxalates. In calciotrophic plants, leaf Ca concentrations are generally higher than leaf potassium concentrations, as is common in the Brassicaceae, Papaveraceae and Resedaceae families, but rare in other plant families. Calcium cations are stored inside cell vacuoles alongside other solutes, including the organic acid anions malate or citrate, which act as both chelators and charge balance for Ca^2+^. The large capacity that Brassicaceae family members have for storing divalent cations in their vacuoles might explain why this family comprises ∼50% of all known metal hyperaccumulator plant taxa (Krämer, 2010). Moreover, the absence of adaptation to extreme osmotic conditions in *A. thaliana* could result from its metabolism, which appears to generally avoid high internal osmotic potentials by storing osmotically inactive starch instead of osmotically active photosynthetic sugars (Streb and Zeeman, 2012). Finally, in association with the high sulfur content of glucosinolates, the Brassicaceae exhibit an active sulfur metabolism and a comparably elevated requirement for sulfate as a nutrient in the soil (Marschner, 1995). More data are required for future comparisons between *A. thaliana* and its sister clade.

## Conclusions

*Arabidopsis thaliana*, a well-established model in molecular genetics and plant cell biology, is progressively being adopted by ecologists and evolutionary biologists, who require far more information on the sites of origin of natural accessions than is contained in the written records of the earlier collections. Insights into the molecular functions of a multitude of individual genes, as well as the elucidation of biosynthetic pathways and regulatory networks in *A. thaliana*, have proven invaluable for identifying the genetic basis of agronomically important traits in crops, such as plant height and flowering time (Doebley et al., 2006; Olsen and Wendel, 2013). The successful transfer of more complex molecular knowledge from *A. thaliana* to crops requires us to account for between-species differences in natural history. Future advances in several disciplines will thus involve, for example, an improved understanding of the diversity of abiotic environments where *A. thaliana* grows naturally, and plant-associated microbial communities, disease and herbivory at these sites (see Box 2). Recently published work highlights the ecological relevance and evolutionary consequences of the relationships between local temperature profiles, plant immunity and hybrid fitness in the field (Chae et al., 2014). In contrast to some endemic and highly specialized plant species among its closest relatives, *A. thaliana* can physiologically acclimate and evolutionarily adapt to a broad range of environments, and the genetic basis underlying this difference is of major interest for future research.

10.7554/eLife.06100.007

Box 2. **Outstanding questions about the natural history of *A. thaliana***

- To understand the genetic diversity between and within populations of *A. thaliana* in the context of their natural environments of origin, with respect to, for example: abiotic factors (including soil composition), plant-associated microbial communities, disease, and herbivory.
- To investigate the natural history of the species closely related to *A. thaliana* for subsequent comparative studies in order to identify the genetic basis of traits that cannot be studied in *A. thaliana.*
- To explore the vast between- and within-species phenotypic variation in the sister clade of *A. thaliana* for the genetic dissection of these traits, and to analyze their ecological roles and the evolutionary history of the underlying polymorphisms. This will additionally provide key insights into the natural history of *A. thaliana.*

**DOI:** http://dx.doi.org/10.7554/eLife.06100.007

However, the natural history of *A. thaliana* is likely to impose some fundamental limitations on its validity as a model for investigating agronomically relevant traits related to the maintenance of growth and survival under stress. Comparative approaches studying *A. thaliana* alongside closely related species will enable us to unravel the molecular basis of traits that are absent in *A. thaliana*, as well as the implications of between-species differences in natural history. Yet our knowledge of the natural history of the species in the sister clade of *A. thaliana* is sketchy at best (see Box 2). Finally, in some cases, it may become relevant to apply the concept of natural history not exclusively at the species level, but also, for example, at the broader genus scale or at the smaller population scale.
